# Research on Joint Resource Allocation for Multibeam Satellite Based on Metaheuristic Algorithms

**DOI:** 10.3390/e24111536

**Published:** 2022-10-26

**Authors:** Wei Gao, Lei Wang, Lianzheng Qu

**Affiliations:** Information and Communication College, National University of Defense Technology, Wuhan 430014, China

**Keywords:** high-throughput satellite system, joint resource allocation, non-dominated beam coding, bandwidth constraint handling, computational complexity, generic application architecture

## Abstract

With the rapid growth of satellite communication demand and the continuous development of high-throughput satellite systems, the satellite resource allocation problem—also called the dynamic resources management (DRM) problem—has become increasingly complex in recent years. The use of metaheuristic algorithms to obtain acceptable optimal solutions has become a hot topic in research and has the potential to be explored further. In particular, the treatment of invalid solutions is the key to algorithm performance. At present, the unused bandwidth allocation (UBA) method is commonly used to address the bandwidth constraint in the DRM problem. However, this method reduces the algorithm’s flexibility in the solution space, diminishes the quality of the optimized solution, and increases the computational complexity. In this paper, we propose a bandwidth constraint handling approach based on the non-dominated beam coding (NDBC) method, which can eliminate the bandwidth overlap constraint in the algorithm’s population evolution and achieve complete bandwidth flexibility in order to increase the quality of the optimal solution while decreasing the computational complexity. We develop a generic application architecture for metaheuristic algorithms using the NDBC method and successfully apply it to four typical algorithms. The results indicate that NDBC can enhance the quality of the optimized solution by 9–33% while simultaneously reducing computational complexity by 9–21%.

## 1. Introduction

Satellite communication systems have rapidly evolved into high-throughput satellite (HTS) systems over the past two decades, with data rates of tens or even hundreds of gigabits per second [1]. In the future, satellite communications will further develop into satellite–terrestrial networks [2], large-scale LEO constellations [3], or 6G networks [4], for which the efficient management of complex resources will be imperative [5]. Owing to the flexibility and complexity of the communication resources allocated in satellite communication systems, it is necessary to develop an automatic tool with efficient algorithms for management of dynamic resources—a problem known as dynamic resource management (DRM) [6]. In response, artificial intelligence algorithms, especially metaheuristic algorithms, are emerging as a fundamental solution to realize fully intelligent resource allocation and management [4]. Studies reported in the literature have focused on four subproblems of the DRM problem: power allocation, bandwidth allocation, joint resource allocation, and beam shape and placement. In addition, the latest research has focused on beamforming [2] and beam hopping [7], as well as their joint allocation with carriers, bandwidth, and power.

The power allocation problem entails maximizing system performance under the limited power of the satellite platform while minimizing total power consumption. The study of power allocation has practical value, as the power resources of satellite platforms are expensive and limited; increased power usage can improve signal gain and enhance user QoS. Choi and Chan [8] characterized the problem of power allocation as a convex optimization problem and introduced Lagrangian multipliers as a solutions, whereas Wang et al. [9] calculated the capacity using satellite link budget equations, which are more practical than the Shannon capacity formula used in [8]. Aravanis et al. [10] demonstrated that power allocation is an NP hard problem and examined the application of metaheuristic algorithms. Using the GA-SA algorithm and the NSGA-II algorithm, the authors proposed a two-stage, multiobjective optimization method to solve the Pareto front. In [11], an allocation model was created, taking interbeam interference and rain attenuation into account and solved using the PSO algorithm. Luis et al. [12] used a deep reinforcement learning (DRL) technique to achieve optimal power allocation in tens of milliseconds; however, the DRL network requires pretraining and could not achieve optimal results in all scenarios. In [13], the performances of GA, SA, PSO, DRL, and hybrid approaches were compared with respect to the power allocation problem. Takahashi et al. [14] combined power allocation with DBF (digital beamforming) to maximize the traffic accommodation rate of an HTS system by jointly optimizing the transmitted power, beam gain, and beam placement.

The bandwidth allocation problem is a subproblem of the frequency assignment problem, which involves assigning the bandwidth of a satellite system to the beam and the frequency subslot of the beam to each user. In [15], it was demonstrated that frequency assignment is an NP complete problem that is difficult to approximate. As a result, some researchers have employed mathematical programming techniques to solve the bandwidth allocation problem. To determine the optimal Lagrange multiplier, Park et al. [16] used a binary search, whereas Heng Wang et al. [17] utilized subgradient algorithms. The aforementioned studies offered solutions for a small number of beams, increasing system capacity or allocation fairness, but are inapplicable to larger datasets. Several studies have been conducted on high-dimensional scenarios. A DRL framework for dynamic channel assignment problems was presented in [18], and a novel image-like tensor reformulation was designed to extract the traffic demand features. In [3], a fast heuristic assignment algorithm for the frequency assignment problem of LEO constellations was proposed, which can achieve the goal of serving a greater number of users with fewer beams.

The joint resource allocation problem is an optimization problem that simultaneously allocates power and bandwidth, typically continuing to develop based on a single resource allocation and achieving increased allocation fairness or communication service capability than single resource allocation. Cocco et al. [19] designed an objective function that combines fairness and demand fit and proposed a stochastic optimization algorithm based on simulated annealing to solve the joint allocation of bandwidth and power. In [20], a link budget model considering interference between beams was proposed based on [10], as well as a GA-based joint resource allocation algorithm, which improved the USC (unmet system capacity) results of the fixed average algorithm by 55.7%. In [21], the PSO-GA and NSGA-II algorithms were used to examine the multiobjective optimization of power and carrier bandwidth for a scenario in which hundreds of beams serve a highly volatile demand. Abdu et al. [22] decomposed the JRA problem into two non-convex subproblems, i.e., carrier and power allocation, and solved them using a continuous convex approximation. He et al. [23] and Gao et al. [24] studied multiobjective optimization with respect to the JRA problem, with the difference that literature [23] used DRL, whereas literature [24] used a metaheuristic multiobjective algorithm, i.e., CMOPSO (multi-objective PSO algorithm with competition mechanism).

In summary, the power allocation and bandwidth allocation problems have been intensively studied, whereas the number of studies on joint resource allocation remains limited. Although DRL [12,18,25] has gradually spread in various satellite resource allocation scenarios, pretraining, generalization, and scenario dependency limit its versatility. Metaheuristic algorithms (e.g., GA, SA, PSO, etc.) are the main methods currently used owing to their speed, ease of encoding, and optimization performance. Nonetheless, the existing research is subject to some limitations: (1) As bandwidth can be used with reuse patterns in multibeam scenarios, any metaheuristic algorithm must deal with overlapping bandwidth constraints. A method called unused bandwidth allocation (UBA) has been proposed in the literature [20] to handle invalid solutions that do not satisfy the bandwidth constraint by repairing the beams one by one, simultaneously increasing the algorithm’s computational complexity and decreasing its exploration capability. (2) Different metaheuristic algorithms have varying convergence times, which are significant in actual operational situations. In addition to the currently studied algorithms, such as GA and PSO, it is necessary to investigate additional metaheuristic algorithms with quicker convergence.

We address these issues by focusing on a typical subproblem of the DRM problem, namely the joint resource allocation problem (JRA) for HTS. Based on the literature [13,20,21,26,27], we construct a link budget model that takes into account interbeam interference and signal modulation patterns, approaching an actual scenario.

Then, a non-dominated beam coding (NDBC) method is proposed to address the shortcomings of the UBA method. We label adjacent rows of beams as dominant and non-dominant beams. The algorithm only encodes and optimizes the bandwidth of the non-dominated beams, and there is no bandwidth overlap constraint between them, such that they can be freely taken in the value domain, which effectively increases the search flexibility and improves the optimization quality of the algorithm. The bandwidths of the dominant beams are then obtained based on the non-dominant beams through a simple calculation. The computational complexity is also reduced, as invalid solutions are no longer repaired.

Finally, the NDBC method is applied to four common metaheuristic algorithms. The simulation results demonstrate that NDBC can effectively reduce the computational complexity while substantially improving the quality of the optimization solutions. We also find that the QPSO algorithm performs better than the GA, DE, and PSO algorithms on the JRA problem. We speculate that QPSO is more sensitive to search flexibility.

The remainder of this paper is structured as follows. In Section 2, we present the link budget model and derive a formula for the beam data rate. In Section 3, we present the unused bandwidth allocation method, propose a non-dominated beam-coding method, and suggest a generic framework for applying the NDBC method to the metaheuristic algorithm. Finally, in Section 4 and Section 5, we analyze the simulation results and conclude this paper, respectively.

## 2. Problem Formulation

### 2.1. Joint Resource Allocation Problem Model

**Figure 1:**
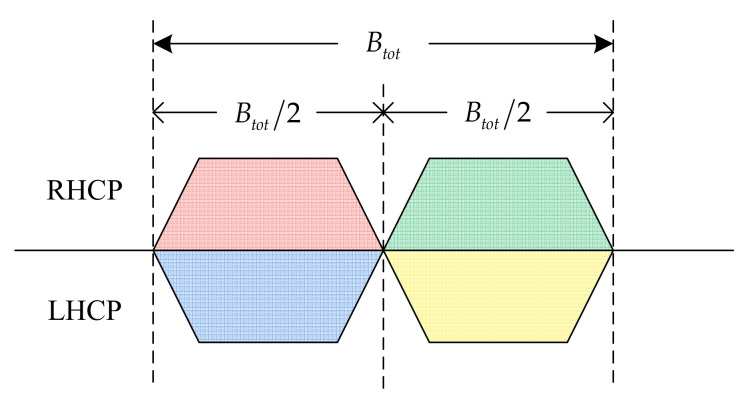
Four-color frequency reuse pattern. Red and green indicate beams using right-hand circular polarization (RHCP), whereas blue and yellow indicate beams using left-hand circular polarization (LHCP).

In this paper, we consider a GEO satellite in the Ka band with M fixed point beams and a multibeam technique in the four-color frequency reuse pattern [1], as depicted in Figure 1. When bandwidth allocation is performed, beams with different polarizations are independent of each other, whereas there is a bandwidth overlap constraint between adjacent beams using the same polarization.

The HTS system can flexibly allocate the bandwidth and power resources of each beam based on the traffic demand of each beam, thereby optimizing QoS and spectrum utilization. The resource allocation (RA) problem, a subset of the JRA problem, has been demonstrated to be an NP hard problem in the literature [10], implying that the JRA problem under consideration in this paper is also an NP hard problem. The objective function of the JRA problem involves minimizing the unmet system capacity (USC), which has been commonly used in the literature [10,20,21]. $D_{i}$ and $R_{i}$ are the traffic demand of the beam and the data rate provided by the system, respectively; $BW_{i}$ and $P_{i}$ are the bandwidth and power resources, respectively, allocated to beam i.

The mathematical description of the JRA problem is as follows:

(1) $$Obj:\min\mathrm{imize}\sum_{i=1}^{M_{b}}\max\left(D_{i}-R_{i},0\right),$$

(2) $$s.t._{}C_{1}:\sum_{i=1}^{M_{b}}P_{i}\leq P_{tot},\forall i\in \left\{1,2,\cdots ,M_{b}\right\}$$

(3) $$C_{2}:P_{i}\leq Pb_{\max},\forall i\in \left\{1,2,\cdots ,M_{b}\right\}$$

(4) $$C_{3}:BW_{i}\leq B_{tot},\forall i\in \left\{1,2,\cdots ,M_{b}\right\}$$

(5) $$C_{4}:BW_{a}+BW_{b}\leq B_{tot},\forall {\left(a,b\right)}_{adj,pol}$$

where $C_{1}$ and $C_{2}$ are the power constraints of the model, typically handled by the proportional reduction method [20]; $C_{3}$ is the bandwidth maximum constraint; and $C_{4}$ indicates that the bandwidths of adjacent beams with the same polarization mode cannot overlap.

### 2.2. Link Budget Model

The carrier-to-noise power ratio of the satellite downlink can be calculated as follows [1,18]:

(6) $$\begin{array}{cc} \left[C/N\right] & =\left[P_{t}\right]+\left[G_{t}\right]-\left[OBO\right]+\left[G_{r}\right]-10\log_{10}\left(T_{sys}\right)\\ & -\left[L_{FS}\right]-\left[L_{O}\right]+228.6-\left[BW\right] \end{array}$$

where $P_{t}$ denotes the transmit power of the satellite antenna (assumed to be numerically equal to the power assigned to the beam); $G_{t}$ and $G_{r}$ denote the gain of the transmit and receive antennas, respectively; OBO is the output backoff factor, taken as a fixed value of 5 dB [20]; $T_{sys}$ represents the receiver system noise temperature, usually at 320 K [1]; $L_{FS}$ denotes the free-space path loss; $L_{O}$ denotes the sum of other losses; and BW is the beam bandwidth.

We consider four primary interference factors, including carrier-to-adjacent-beam interference (CABI), carrier-to-adjacent-satellite interference (CASI), carrier-to-cross-polarization interferences (CXPI), and carrier-to-third-order intermodulation product interference (CIMI) [20]. Then, taking interference into account, the carrier-to-noise ratio is [27]:

(7) $$\begin{array}{c} {\left[\frac{C}{N+I}\right]}^{-1}={\left[\frac{C}{N}\right]}^{-1}+{\left[\frac{C}{I}\right]}^{-1}=\\ {\left[\frac{C}{N}\right]}^{-1}+{\left[\frac{C}{ABI}\right]}^{-1}+{\left[\frac{C}{ASI}\right]}^{-1}+{\left[\frac{C}{XPI}\right]}^{-1}+{\left[\frac{C}{IMI}\right]}^{-1} \end{array}$$

where $E_{b}$ denotes the bit energy; C is the carrier power; and $R_{b}$ represents the data rate (i.e., the bit rate, in bps), satisfying the relation $R_{b}=1/T_{b}$, where $T_{b}$ denotes the bit duration and the relationship between the signal bit energy and the carrier power (i.e., $E_{b}=CT_{b}$). We assume that the noise bandwidth is equal to the beam bandwidth. Therefore, the density ratio of energy per bit to noise plus interference is calculated as follows:

(8) $$\frac{E_{b}}{N_{0}+I_{0}}=\frac{CT_{b}}{N_{0}+I_{0}}=(\frac{C}{N_{0}+I_{0}})\times \frac{B_{N}}{R_{b}B_{N}}=(\frac{C}{N+I})\frac{BW}{R_{b}}.$$

The signal is modulated with a 4/8/16/32/64 element code, with the coding rate taking values from 1/4 to 9/10. Hence, the ratio of energy per symbol to noise plus interference power spectral density is [26]:

(9) $$\frac{E_{s}}{N_{0}+I_{0}}=\frac{E_{b}r\log_{2}M}{N_{0}+I_{0}}=(\frac{C}{N+I})\frac{BW}{R_{b}}r\log_{2}M.$$

Typically, such a system employs an ACM (adaptive coding and modulation) strategy to maximize spectral efficiency. According to [12], the beam data rate is:

(10) $$R_{b}=R_{s}\times \Gamma (\frac{E_{s}}{N_{0}})=\frac{BW}{1+\alpha_{r}}\times \Gamma (\frac{E_{s}}{N_{0}}),$$

where $\alpha_{r}$ denotes the roll-off factor, $R_{s}$ denotes the symbol rate, and Γ is the spectral efficiency (in bit/symbol) of the modulation and coding scheme (MODCOD), satisfying the following conditions [13]:

(11) $$\frac{E_{s}}{N_{0}+I_{0}}\geq {\left.\frac{E_{s}}{N_{0}}\right|}_{MODCOD}+\mu ,$$

where μ is the desired margin of the link (specified as 1.0 dB), whereas ${\left.E_{s}/N_{0}\right|}_{MODCOD}$ denotes the threshold value required for the MODCOD scheme employed by the link to obtain the spectral efficiency (Γ) under ideal channel conditions. Accordingly, to calculate the data rate, we assume a certain MODCOD mode, then use Equations (6)–(10) to calculate $E_{s}/\left(N_{0}+I_{0}\right)$. Then, we check whether condition (11) is satisfied, thus maximizing the spectral efficiency of the link to the greatest extent possible.

## 3. Constraint Handling Method

### 3.1. Unused Bandwidth Allocation

The metaheuristic algorithm contains operators with randomness, which cause some individuals not to satisfy the constraints and become invalid solutions. Solutions to deal with invalid solutions often include discarding, repairing, penalizing, and transferring; the UBA described below uses the repair method.

**Figure 2:**
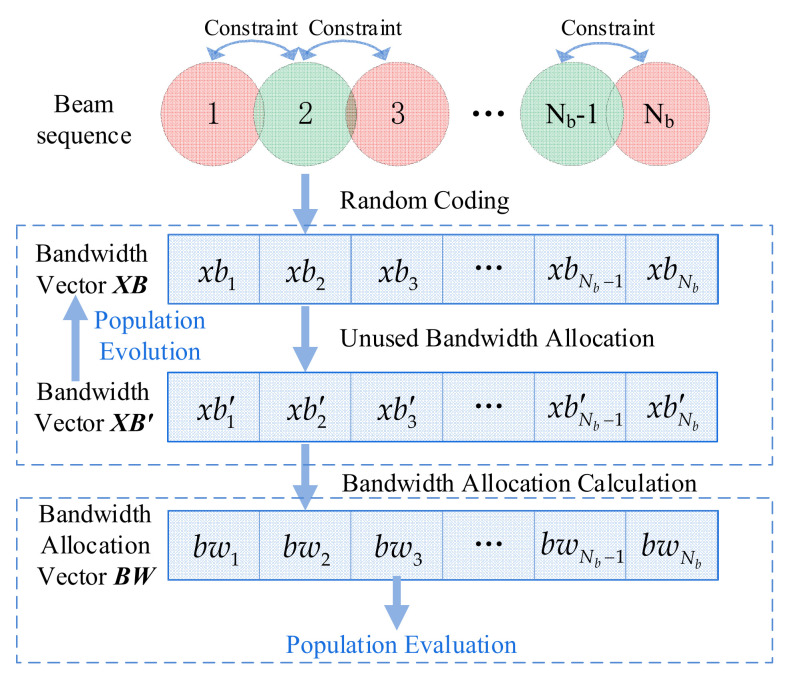
Principles of coding methods and algorithm evolution (UBA).

As depicted in Figure 2, the unused bandwidth allocation (UBA) method [20] is utilized primarily to address the bandwidth constraint of invalid solutions. When using UBA, all beams are encoded and optimized, such that all neighboring beam pairs have bandwidth overlap constraints.

We assume a sequence of adjacent beams with $N_{b}$ beams; a corresponding individual in the algorithm is $X=\left[XP,XB\right]$, where XP and XB denote the power vector and the bandwidth vector, respectively. The bandwidth constraint can be handled as follows:

(1) With a probability of 0.5, the starting beam is selected as 1 or $N_{b}$. The beam pair is expressed as follows:
  (12) $$\left(beam_{a},beam_{b}\right),b=a+1,a\in \left\{1,2,\cdots ,N_{b}-1\right\},$$
  (13) $$\left(beam_{a},beam_{b}\right),b=a-1,a\in \left\{N_{b},N_{b}-1,\cdots ,2\right\}.$$
(2) For each pair of beams $\left(beam_{a},beam_{b}\right)$, if $xb_{a}+xb_{b}>1$ is satisfied, the operation $xb_{b}=1-xb_{a}$ is performed in order to ensure that constraint $C_{4}$ is satisfied; however, some bandwidth may be unused.

**Figure 3:**
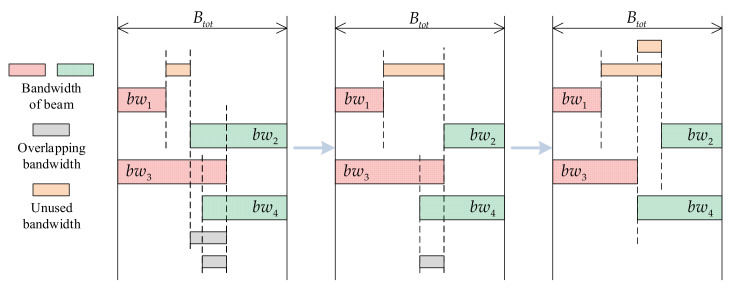
Elimination of overlapping bandwidth and the emergence of unused bandwidth.

Using a column of four adjacent beams as an example, Figure 3 illustrates the underlying principle of this step. As bandwidth overlap is eliminated, unused bandwidth is gradually generated and expanded. Consequently, the bandwidth of some beams is altered; this phenomenon has a detrimental effect on the search direction of the algorithm. It is possible that when the beam’s bandwidth is increased to improve the objective function, a smaller value is obtained after this step (and vice versa), causing the algorithm to search in the opposite direction.

(3) The beams are ordered from largest to smallest based on the demand. The serial number sequence is denoted as SortD.
  (14) $$b_{unused}=\left\{\begin{array}{l} 1-xb_{i}-\max\left(xb_{L},xb_{R}\right)\\ 1-xb_{i}-xb_{A} \end{array}\right.,$$
  (15) $$x{b^{\prime }}_{i}=xb_{i}+b_{unused},i\in SortD,$$
  where $xb_{L}$ and $xb_{R}$ denote the bandwidths of the adjacent beams when beam i has two adjacent beams, whereas $xb_{A}$ is the bandwidth of the adjacent beam when beam i has only one adjacent beam. As shown in Figure 2, for population evaluation, the bandwidth vector must be transformed into a bandwidth allocation vector:
  (16) $$bw_{i}=x{b^{\prime }}_{i}\times B_{tot},i\in \left\{1,2,\cdots ,N_{b}\right\}.$$

The UBA method has two issues: First, in step 2, it needs to repair the positions of a significant number of particles in the algorithm population while only ensuring that this portion of particles satisfies the constraint rather than having performed a valid search. These particles do useless work with greater probability, in addition to increasing the computational complexity. Second, the low-bandwidth flexibility of each beam affects the algorithm’s ability to search the solution space, reducing its global optimization capability.

### 3.2. Non-Dominated Beam Coding

We assume that beams with different polarizations are independent; therefore, we focus on the bandwidth overlap between adjacent beams with the same polarization. We assume that beam B dominates beam A when beam A is adjacent to beam B and that the bandwidth of beam A is influenced by beam B. If we treat the bandwidths of the beams sequentially, the dominance phenomenon is almost ubiquitous; however, if we assign values to the bandwidth beams at intervals, there is no influence between them.

**Figure 4:**
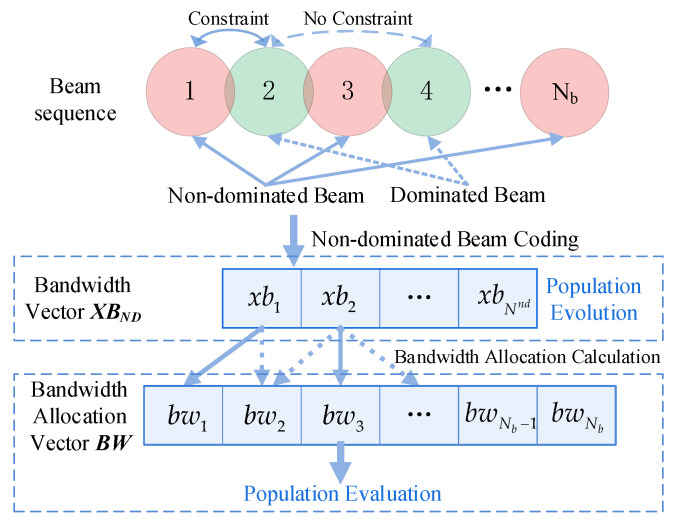
Principles of coding methods and algorithm evolution (NDBC). A column of adjacent beams is set up one by one as a dominant beam column and a non-dominant beam column, respectively. The two beam columns are denoted as $BS_{D}=\left\{2,4,\cdots \right\}$ and $BS_{ND}=\left\{1,3,\cdots ,N_{b}\right\}$, with $N^{d}$ and $N^{nd}$ beams, respectively.

As depicted in Figure 4, the adjacent non-dominant beams dominate the bandwidth of the dominant beam, and there is no bandwidth overlap constraint between non-dominant beams. The bandwidth vector ($XB_{ND}$) can be encoded as follows:

(17) $$xb_{j}=r,r\sim U\left(0,1\right),j\in BS_{ND},$$

(18) $$XB_{ND}=\left[xb_{1},xb_{2},\cdots ,xb_{N^{nd}}\right].$$

**Figure 5:**
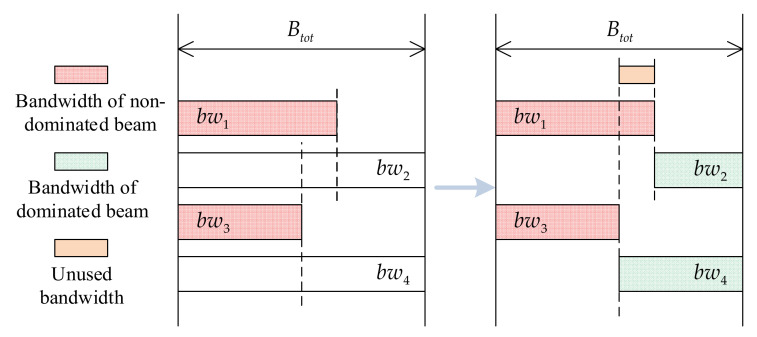
Calculation of the bandwidth of the dominant beam. To maximize bandwidth utilization, we set bandwidth of the dominant beam to its maximum value (subject to the constraints). Although unused bandwidth remains, this bandwidth cannot be reallocated in the current scenario.

As demonstrated in Figure 5, the NDBC method encodes only the non-dominated beams and generates directly corresponding bandwidth values, whereas the bandwidth of the dominated beams is determined by their respective adjacent dominated beams.

Once the population has evolved during the population evolution session, it must be converted to a bandwidth allocation vector in order to ensure that the bandwidth constraint is met. The conversion method is as follows:

(19) $$bw_{j}=xb_{i}\times B_{tot},\forall j=nd_{i}\in BS_{ND},i\in \left\{1,2,\cdots ,N^{nd}\right\},$$

(20) $$bw_{j}=\left\{\begin{array}{l} \left[1-\max\left(xb_{a},xb_{b}\right)\right]\times B_{tot},\exists {\left(a,j,b\right)}_{adj,p}\\ \left(1-xb_{a}\right)\times B_{tot},only_{}\exists {\left(a,j\right)}_{adj,p} \end{array}\right.,for\left\{\begin{array}{l} \forall j\notin BS_{ND}\\ \forall a,b\in BS_{ND}\&a\neq b \end{array}\right..$$

### 3.3. Complexity Analysis and General Application Architecture

Assume that M denotes the total number of beams, $G_{e}$ represents the number of generations, and $N_{pop}$ is the algorithm’s population size. For the UBA method, the computational complexities associated with eliminating overlapping bandwidth and allocating unused bandwidth are $O\left(G_{e}N_{pop}M\right)$ and $O\left(G_{e}N_{pop}M\right)$, respectively. The total computational complexity is as follows:

(21) $$O\left(G_{e}N_{pop}M+G_{e}N_{pop}M\right)=O\left(2G_{e}N_{pop}M\right).$$

For the NDBC method, the computational complexity of the method is primarily attributable to the computation of the dominant beam’s bandwidth. We assume $M^{nd}$ as the total number of non-dominated beams. The computational complexity is as follows:

(22) $$O\left(G_{e}N_{pop}M^{nd}\right)\approx O\left(\frac{1}{2}G_{e}N_{pop}M\right).$$

Therefore, the computational complexity of NDBC is approximately one-fourth that of UBA. As shown in Table 1, we investigated the degree of beam dominance for UBA and NDBC in order to determine the effect of the bandwidth constraint handling method on the search flexibility of the algorithm. As the order is randomly reversed in step 1 for UBA, the first and last beams are actually dominated by their neighboring beams. The non-dominated beams in NDBC are not dominated by one another, whereas the first and last beams are dominated by only one of their neighboring beams. We quantify the number of neighboring beams that can exert influence on a beam as its degree of dominance; the greater the degree of dominance, the less flexible the beam’s bandwidth.

As shown in Table 1, assuming a column of adjacent beams with M beams, the UBA method is dominated by two adjacent beams for almost all beams. On the other hand, NDBC only needs to encode and optimize the bandwidth of the non-dominated beams, thereby practically achieving complete search flexibility. This modification effectively enhances the algorithm’s global optimization performance. The general architecture of the NDBC method applied to the metaheuristic algorithm is depicted in Table 2.

## 4. Results

**Figure 6:**
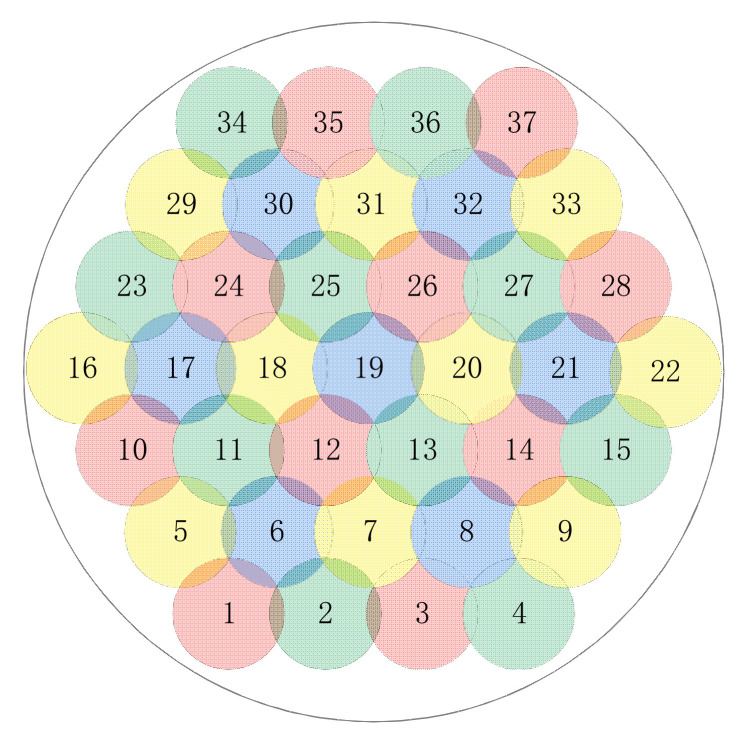
Schematic diagram of the distribution of 37 beams. Using the NDBC method, all red and blue beams (17 in total) are selected as non-dominated beams, whereas all green and yellow beams (20 in total) are dominated beams. The total demand for traffic across all beams is 24.16 Gbps, with a standard deviation of 177 Mbps.

The simulation scenario was configured as a GEO multibeam satellite with 37 beams. Figure 6 depicts the beam distribution. The majority of the simulation scenario parameters were obtained from [1,20]. Table 3 details some of the most important parameters.

Standard metaheuristic algorithms, such as GA [20], DE [28], PSO [29], and QPSO [30], were utilized in our simulations. For a fair comparison, each algorithm’s population size and termination conditions were set to the maximum number of iterations. Table 4 provides the algorithm parameters. A DVB-S2 [31] and DVB-S2X [32] hybrid MODCOD table was used, which was screened such that as the threshold value increased, the spectral efficiency increases uniformly.

**Figure 7:**
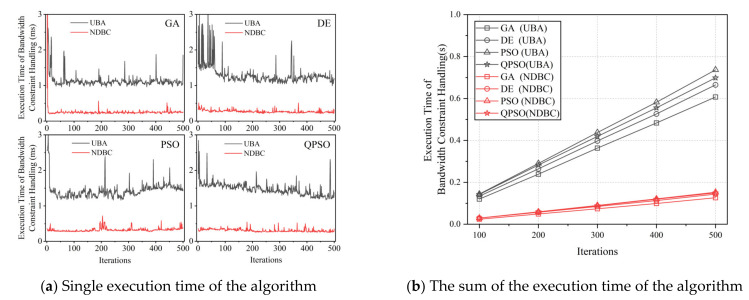
The execution time required for the constraint handling session: (**a**) constraint handling time of a single execution (with 500 iterations and a population size of 400); (**b**) total constraint handling time generated by both methods versus the number of iterations (with a population size of 400). The results in the graph demonstrated that NDBC can reduce the execution time of the bandwidth-constrained handling session by 75–80%.

As depicted in Figure 7, the execution time of the constraint handling session varied across iterations due to the random nature of the algorithm’s evolution. NDBC was more stable and required less time to execute each algorithm than UBA. The execution time of constraint handling is proportional to the number of iterations. Quantitatively, NDBC required approximately one-fourth to one-fifth of the time required by UBA, achieving an effective reduction, indicating that the computational complexity of NDBC is lower than that of UBA. This result is consistent with the analysis presented in Section 3.3 regarding computational complexity.

**Figure 8:**
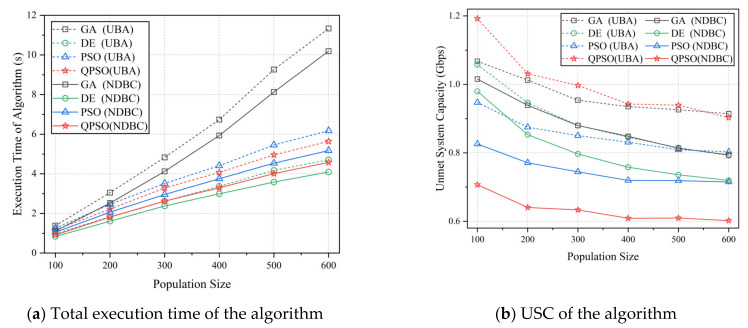
The execution time (**a**) and optimized solutions (**b**) obtained by the algorithms. The population size of each algorithm ranged from 100 to 600, and 300 iterations were performed. The dashed and solid lines in the figures represent the UBA and NDBC methods, respectively, whereas different colors represent different algorithms.

The optimal results of each algorithm with UBA and NDBC are depicted in Figure 8. According to the model presented in this paper, the USC was 1.967 Gbps when bandwidth and power are distributed uniformly (denoted as average bandwidth and average power allocation, ABAP). Figure 8a illustrates the execution time of each algorithm for varying population sizes. Except for the GA, the relationship between the algorithm’s execution time and population size was approximately linear. The DE, PSO, and QPSO algorithms executed faster than the GA algorithm. In comparison to the UBA method, the NDBC method reduced the total execution time of each algorithm by 9–21%. Figure 8b depicts the mean values of each algorithm’s optimized solutions for varying population sizes. The quality of the optimal solution improved proportionally to the size of the population. As depicted in the graph, the NDBC method significantly improved the optimized solution of each algorithm by 9–33%. In terms of algorithm comparison, the strength of algorithm optimization capability is ranked as QPSO, PSO, DE, and GA. However, when using UBA, this ordering is PSO, DE, GA, and QPSO. We speculate that QPSO is more sensitive to the flexibility of the search compared to other algorithms.

Increasing the population size can result in a more optimal solution, but it also increases the execution time. In the following simulations, the population size was set to 400 in order to control the execution time of the algorithm without causing it to take too long. The maximum and minimum number of iterations were 500 and 200, respectively. After 50 iterations, the algorithm terminated if the improvement in the optimized solution was less than $5\times 10^{-5}$.

**Figure 9:**
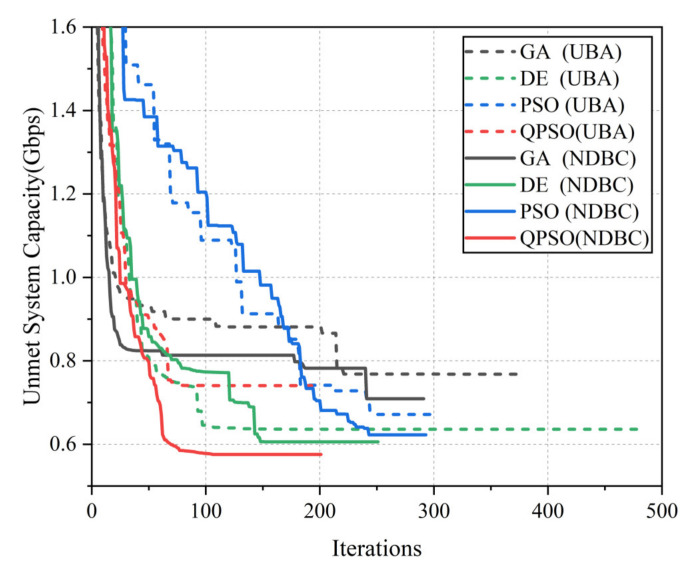
Convergence for the best execution of the algorithms using different bandwidth constraint handling methods. The dashed and solid lines in the figure represent the UBA and NDBC methods, respectively, whereas different colors represent different algorithms.

Figure 9 depicts the convergence curves with the best results from 50 executions of each algorithm under the threshold termination condition, reflecting the convergence speed and optimization capability of each algorithm. As depicted in Figure 9, GA and QPSO had faster convergence rates, reaching convergence after approximately 100 iterations. The NDBC method enhanced the quality of each algorithm’s optimized solution and accelerated their convergence, and DE and QPSO showed greater potential. In combination with the algorithm execution time, QPSO can achieve the best optimized solution in the shortest time, indicating that QPSO performs better when the search flexibility is high.

As shown in Table 5, the improvement in GA (UBA) in the mean USC metric (0.958) was 51% compared to UBUP (1.967), as opposed to 55.7% (1.9→0.84) reported in the literature [20]. We modified the link budget model based on [20] and used a different MODCOD table such that the results slightly differed. The best average USC result of the QPSO algorithm utilizing NDBC was 0.641, representing a 67.4% improvement relative to UBUP (1.967) and a 21% improvement relative to the result reported in [20]. The NDBC method improved all USC metrics in comparison to the UBA method, with improvements of 9–36% for the USC mean and 14–38% for the execution time.

**Figure 10:**
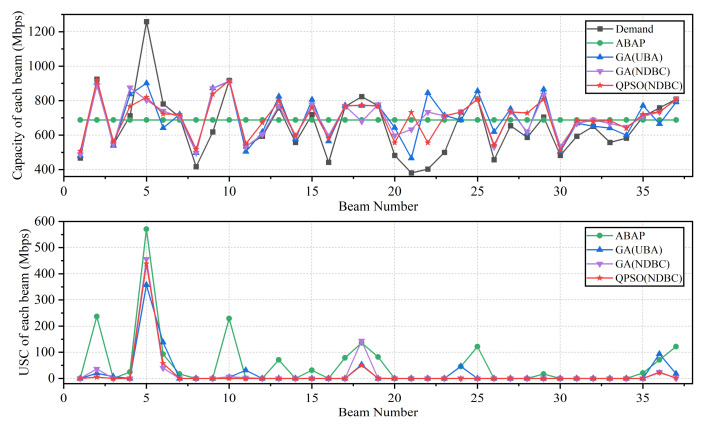
Capacity allocation and USC of each beam for the best single execution of the algorithm.

For an efficient comparative analysis, we selected the ABAP algorithm, the GA(UBA) scheme from [20], the comparison algorithm GA(NDBC), and the QPSO(NDBC) algorithm, which applied NDBC most effectively. Figure 10 depicts the capacity allocation and USC outcomes for each beam based on the four previously mentioned algorithms. Using the GA algorithm, NDBC was able to achieve better allocation results for beams 6, 11, 18, 24, and 36 in comparison to UBA. QPSO was able to improve the USC results in beams 2 and 18 using NDBC in comparison to GA. Overall, NDBC reduced resource allocation for high-demand beams, improved USC, and increased allocation fairness.

An important aspect of satellite resource allocation, i.e., traffic demand, depends on the specific geographical location and the associated time of day; therefore, when considering a large footprint composed of multiple beams (as in this paper), with beams experiencing varying capacity requests that also vary in time (slowly, i.e., on the order of hours). In this line, resource allocation in continuous time scenarios has been researched in various studies [12,18,19]. Simulation experiments were subsequently conducted in order to determine whether the application of the NDBC method in the continuous time case deviated from the results in the static scenario.

**Figure 11:**
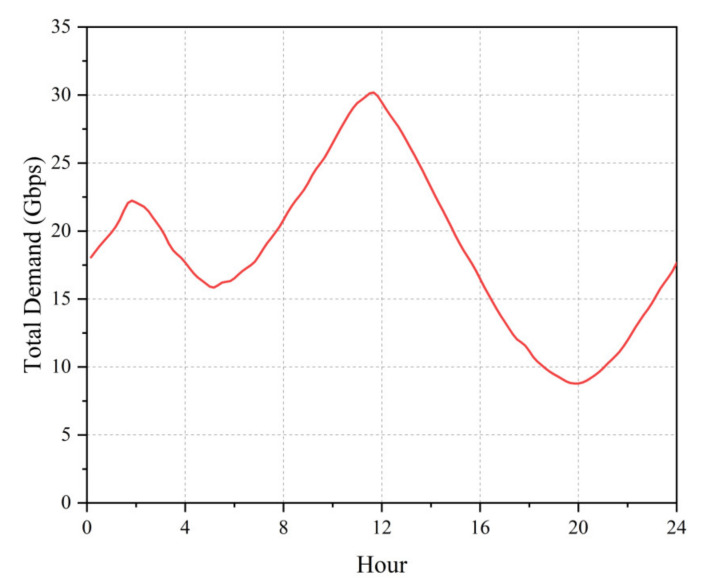
Evolution of total demand over time.

Assuming that the geographical characteristics that influence beam demand remain constant, the demand is primarily influenced by temporal characteristics. The demand ratio of each point beam was considered to be identical to the stationary scenario described previously, with a random deviation of 20% or less added to simulate the actual situation. Figure 11 illustrates the total demand, with a sampling interval of 10 min and a scheduling time limit of 3 s. The maximum and minimum number of iterations was 500 and 200, respectively. After 50 iterations, the algorithm terminated if the improvement in the optimized solution was less than $5\times 10^{-5}$.

**Figure 12:**
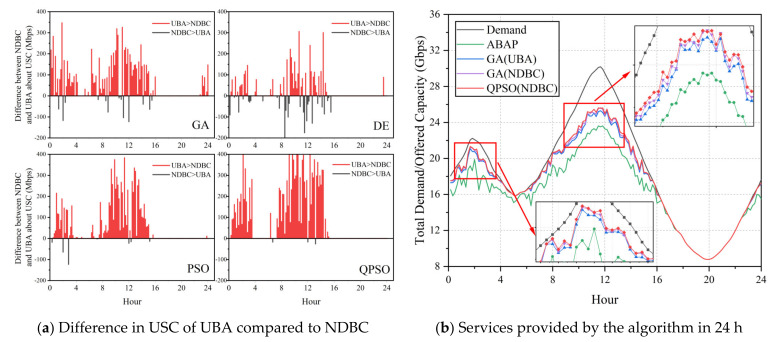
The execution time and optimized solutions of the algorithms in continuous-time demand scenarios. (**a**) The red vertical line indicates where the USC of NDBC is better than that with UBA, and vice versa for grey; and (**b**) the actual offered capacity calculated by subtracting the USC value from the total required capacity at each instant (this value is usually not equal to the total allocated capacity).

As shown in Figure 12a, the USC values of NDBC for all algorithms were superior to those of UBA in the majority of instances, whereas the application of the QPSO and PSO algorithms provided superior results compared to GA and DE. In conjunction with Table 5, it is evident that the stability of PSO and QPSO utilizing NDBC is superior to that of GA and DE (the standard deviations of the average USC were 46, 46, 65, and 64, respectively). We selected the average allocation algorithm (abbreviated as ABAP), the scheme GA(UBA) from [20], the comparison algorithm GA(NDBC), and the QPSO algorithm applying NDBC, with the best results, in order to investigate the service capability of the four aforementioned algorithms in the continuous demand scenario.

Figure 12b depicts the following phenomena: (1) the metaheuristic algorithm improved the serviceability of the fixed-average algorithm, (2) NDBC can improve the service capability of UBA when the algorithm is GA, and (3) when using NDBC, QPSO can achieve a better solution than GA. Owing to the limited resources, the system service capacity reaches the upper session limit of its service capacity during the peak demand period of the day. In general, the NDBC method proposed in this paper can adapt to continuous-time scenarios.

## 5. Conclusions

In this paper, we proposed a non-dominated beam-coding (NDBC) method to solve the adjacent bandwidth overlap constraint in the HTS joint resource allocation problem, achieving full bandwidth flexibility in the evolution of the algorithm while decreasing computational complexity. By applying the NDBC method to a metaheuristic algorithm, it is possible to reduce the algorithm’s execution time while simultaneously enhancing the quality of the resource allocation results. Finally, a general architecture for applying NDBC to metaheuristic algorithms was developed. Simulations demonstrated that NDBC can reduce the computational complexity of the bandwidth-constrained handling session by 75–80% and the overall algorithm execution time by 9–21% while improving the USC results by 9–33%. We also found that when NDBC was applied, the PSO and QPSO algorithms performed better than the GA and DE algorithms. Importantly, the model does not consider all aspects of a multibeam satellite communication system, as doing so would make the problem extremely complex and difficult to describe. Our conclusions remain unaffected by factors not accounted for in the model, such as the antenna pattern, satellite payload characteristics, overall frequency plan (feeder and user links), rain attenuation, and precoding. The results reported herein are instructive for the application of metaheuristic algorithms to multibeam satellite DRM problems.

## Figures and Tables

**Table 1:** Domination degree of UBA and NDBC.

Degree of Domination	UBA	NDBC
0	0	≈M/2
1	2	2
2	M−2	≈M/2−2

**Table 2:** The general architecture of the metaheuristic algorithm using the NDBC method.

**Input:**D(Demand); **Output:**$P_{best}$**,**$BW_{best}$**,**$R_{best}$
1: /*Initialization*/ 2: /*Non-dominated Beam Coding*/ 3: **while** iteration < Maximum iterations 4: /* Population Evolution */ 5: **for** each operator in Algorithm do 6: /*Population Evolution*/ 7: /*Power Constraint Handling*/ 8: **end for** 9: /*Population Evaluation*/ 10: /*Bandwidth Constraint Handling*/ 11: /*Dominated Beam Bandwidth Calculation*/ 12: /*Beam Data Rate Calculation*/ 13: /*Get Optimal Solution*/ 14: **end while** 15: **Return** the final solution

**Table 3:** Parameters used in the simulation scenario.

Parameter	Symbol	Value	Unit
Total power	$P_{tot}$	2350	W
Total bandwidth	$B_{tot}$	375 (×2)	MHz
Power maximum	$Pb_{\max}$	100	W
Transmit antenna gain	$G_{t}$	52.2	dB
Receive antenna gain	$G_{r}$	41.5	dB
Free-space path losses	$L_{FS}$	209	dB
System temperature	$T_{sys}$	320	K
Roll-off factor	$\alpha_{r}$	0.2	—
Link margin	μ	1.0	dB
Carrier-to-adjacent-beam interference	C/ABI	36	dB
Carrier-to-adjacent-satellite interference	C/ASI	28	dB
Carrier-to-cross-polarization interference	C/XPI	30	dB
Carrier-to-third-order inter-modulation interference	C/IMI	21	dB

**Table 4:** Algorithm parameters.

GA	GA	DE	DE
Parameter	Value	Parameter	Value
Tournament size Blend Alpha Crossover prob. Mutation prob.	5 0.2 0.95 [0.05, 0.15]	Ini. Mutation Prob. Crossover Prob.	0.2 0.1
**PSO**	**PSO**	**QPSO**	**QPSO**
**Parameter**	**Value**	**Parameter**	**Value**
Inertia weight Cognitive factor Social factor	[0.2, 1.0] 1.0 1.0	Systolic-expansion factor	[0.01, 0.8]

**Table 5:** Optimal solution and execution time of the algorithm.

Algorithm	Result	GA	DE	PSO	QPSO
UBA	Worst run	1.115	1.016	0.986	1.538
	Average run	0.958	0.826	0.807	1.006
	Best run	0.768	0.636	0.671	0.741
	Standard deviation	62.28	73.28	45.16	115.21
	Average execution time	5.16	4.39	4.16	2.77
	Average iteration	248.96	423.58	314.27	216.39
NDBC	Worst run	1.026	0.912	0.853	0.798
	Average run	0.866	0.749	0.686	0.641
	Best run	0.709	0.606	0.623	0.576
	Standard deviation	64.53	65.65	46.74	46.80
	Average execution time	4.02	2.66	3.57	2.23
	Average iteration	220.02	292.45	321.13	214.46

## Data Availability

Data are contained within the article.
